# Recent Progress on Luminescent Metal-Organic Framework-Involved Hybrid Materials for Rapid Determination of Contaminants in Environment and Food

**DOI:** 10.3390/polym12030691

**Published:** 2020-03-20

**Authors:** Chi-Xuan Yao, Ning Zhao, Ji-Chao Liu, Li-Jun Chen, Jing-Min Liu, Guo-Zhen Fang, Shuo Wang

**Affiliations:** 1 State Key Laboratory of Food Nutrition and Safety, Tianjin University of Science & Technology, Tianjin 300457, China; yaochixuan@mail.tust.edu.cn (C.-X.Y.); fangguozhen@tust.edu.cn (G.-Z.F.); 2 Tianjin Key Laboratory of Food Science and Health, School of Medicine, Nankai University, Tianjin 300071, China; 15891783438@163.com (N.Z.); liujingmin@nankai.edu.cn (J.-M.L.); 3 Beijing San Yuan foods co., LTD., No. 8 Yingchang Road, Yinghai, Daxing District, Beijing 100076, China; lichunyanglucy@163.com

**Keywords:** metal-organic frameworks, fluorescence, contaminants, food, environment

## Abstract

The high speed of contaminants growth needs the burgeoning of new analytical techniques to keep up with the continuous demand for monitoring and legislation on food safety and environmental pollution control. Metal-organic frameworks (MOFs) are a kind of advanced crystal porous materials with controllable apertures, which are self-assembled by organic ligands and inorganic metal nodes. They have the merits of large specific surface areas, high porosity and the diversity of structures and functions. Latterly, the utilization of metal-organic frameworks has attracted much attention in environmental protection and the food industry. MOFs have exhibited great value as sensing materials for many targets. Among many sensing methods, fluorometric sensing is one of the widely studied methods in the detection of harmful substances in food and environmental samples. Fluorometric detection based on MOFs and its functional materials is currently one of the most key research subjects in the food and environmental fields. It has gradually become a hot research direction to construct the highly sensitive rapid sensors to detect harmful substances in the food matrix based on metal-organic frameworks. In this paper, we introduced the synthesis and detection application characteristics (absorption, fluorescence, etc.) of metal-organic frameworks. We summarized their applications in the MOFs-based fluorometric detection of harmful substances in food and water over the past few years. The harmful substances mainly include heavy metals, organic pollutants and other small molecules, etc. On this basis, the future development and possible application of the MOFs have prospected in this review paper.

## 1. Introduction

### 1.1. The Harmful Substance for Health

In recent years, with the continuous population growth and industrial and societal development, the security issues caused by environmental and food pollution have become the fateful consequences for the ecosystem and public health. Food safety is closely related to environmental pollution. Environmental contaminants can be enriched to toxic concentrations through terrestrial and aquatic food chains [1]. The joint action on the increasing risk of food safety and environmental pollution has affected a large part of the population in the world. Water scarcity, heavy metals, environmental organic compounds and chemical pollutants, disfavored farming and animal husbandry, impacted on food safety and threatened human health [2,3]. The excretion of human or veterinary pharmaceuticals turned out to be environmental contaminants due to insufficient elimination during sewerage in sewage treatment plants [4,5,6]. These contaminants were brought to food products in the process, including but not limited to the raw material of food (animals and plants via the water, soil or feed), processing, preservation, or packaging [3,7]. Since these contaminants can be devastating to the aquatic environment, considerable attention has been focused on their detection and assessment in waste- and surface-water.

On the other hand, technical progress has also allowed a dramatic increase in output for modern agriculture. Every link of the agricultural and food industry system is manifested as by-products, waste, and air emissions with a possible impact on the environment [8,9]. Some studies have been published about the environmental effects of sustainable food production and food consumption with their related processes [10,11]. On the other hand, food trash has a relationship with food preservation and packaging, inferior quality food products, food contamination and spoilage. Food trash is of great significance on the globalization of environmental, social and economic implications.

The problems that arise from above are inextricably bound up with the natural environment. Some toxic and harmful substances coexisted widely in the environment and food. People come to realize that the highly sensitive, accurate and convenient analytical procedures to detect contaminants are extremely important in food safety and environmental analyses.

### 1.2. The Introduction of Metal-Organic Frameworks

Metal-organic frameworks (MOFs), also known as porous coordination polymers (PCPs), are the porous network materials constituting of nodes (metal ions or clusters) and bonds (organic ligands) to connect and extend joints [12]. These kind of coordination polymers were invented in 1960 however marked expansion in this domain has been obtained basically from 1995 when Yaghi create the concept of “metal-organic frameworks” [13]. MOFs were also used in the research fields of hybrid polymer species frequently. The composition of nanocomposites between MOFs and polymers provided the unique platforms to achieve the nanoscale functions such as controlling polyreaction of polymers, new MOF-polymers hybrid membranes and stabilization of immiscible polymer blends using MOFs to give the new materials with pioneering features which were not realizable with the single-handed components. The distinctions between the characters of MOFs and these polymers has positive influence to cross luminescent metal-organic frameworks (LMOFs) and multifarious polymers to produce composites to keep required properties of these distinct materials. Remarkably, the studies have shown that MOFs can be intended for influencing the structures of polymers, and polymers can be used to regulate the growth and characteristics of MOFs. Moreover, the design and manufacture of MOF hybrid materials with polymers is of considerable interest to improve the stability and functionality of MOF materials in sensing for the undetected contaminants of the food and environment.

MOFs have an infinitely extended periodic frame structure, in which the coordination bond between metal and ligand is stronger than that of hydrogen bond, and there are also weaker interactions than hydrogen bond, such as π-π interaction. The unique structures allow MOFs the good performances of excellent utilization in adsorption, separation, storage, catalysis and controlled release [14,15]. Over 20,000 categories of MOF materials have been reported so far. Compared with traditional porous materials, metal-organic materials appeared as advanced porous material in the field of food and environment. For example, (i) MOFs were abundant in the advantages of wide varieties, diversity of properties; (ii) MOFs were provided with highly tunable pores for analytes with specific size and shape [16], both the inorganic metals and ligands of MOFs could be adjustable for different purposes, so that MOFs with different porosity and pore structures can be used in many various applications [17,18,19,20]; (iii) MOFs can be simply introduced into the functional groups with durable functionality; (iv) MOFs with different structures can be synthesized by different organic ligands, and additionally, various functional groups can also be introduced through the modification after synthesis to realize the functionalization of MOFs; (v) the stable rigid structures of MOFs could help MOFs to endure harsh environments, such as wide pH ranges, and high ionic strengths and temperatures. The stability creates conditions for the recyclability of MOFs and especially plays a vital role as sorbents for MOFs in the adsorption process. MOFs usually possess hollow structures so that this provided them the larger specific surface area and lower density. The detection of harmful substances is an essential content in the field of food safety. It should put forward high requirements for its detection method due to the low level of deleterious residues [21] and the complicated matrix of the dairy products samples.

Luminescent metal-organic frameworks (LMOFs), which are the members of metal-organic frameworks with relevant fluorescent structures, have been a research hotspot due to their tunable photoluminescence properties. The LMOFs as same as structural diversity of MOFs can also yield countless applications. The luminescent characteristics of were closely related to numerous factors, including the components of synthetic raw materials, the coordination environment of the metal nodes, chemical structure/volume of the pores, and host-guest interactions between the LMOFs and reacting types (e.g., non-covalent interactions, coordination bonds, and π-π interactions). The combined power of these determinants proves the enlargement of their application in numerous fields accurately.

The synthesis of MOFs refers to the self-assembly of organic ligands and metal ions or metal ion clusters to form three-dimensional grid crystals [22]. In summary, there are four main ways to synthesize MOFs materials.

#### 1.2.1. The Solvothermal Method

MOFs were synthesized by adding organic ligands and metal ion sources into water or organic solvents and heating the reaction system. According to different heating methods, it can be divided into the electric heating method [23,24], the microwave method [25,26], the ultrasonic method [27]. Compared with the electrical heating method, the microwave method and the ultrasonic method significantly increased the reaction rates and greatly reduced the reaction time. Wang [28] reported two luminescent MOFs with the same ligand synthesized by the solvothermal method to assess the nitroaromatic explosives and DNA strands. Zhou [29] reported a red luminescence MOF for LED application using the microwave method. Hu [27] published a facile and environment-friendly synthesis of luminescent MOF nanowires using the ultrasonic method for fluorescence-based aromatic amines detection. To a certain extent, this simple and direct method enables scientists to study in their respective fields more easily and conveniently. This method also promotes the screening and discovery of new materials in a short period of time.

#### 1.2.2. The Mechanochemical Synthesis Method

The mechanochemical methods have emerged recently which can replace alternative solvothermal method, including solid-state and liquid-assisted grinding and ultrasound-assisted grinding. Compared with solvothermal synthesis, these methods have many apparent advantages, such as free of organic solvents, high yield, and low environmental pollution due to recycling of oxidant [30]. For example, some scholars use the mechanochemical methods to synthesize HKUST-1 (Cu_3_(BTC)_2_, BTC = trimesic acid) and MOF-14 (Cu_3_(BTB)_2_, BTB = 1,3,5-Tris (4-carboxyphenyl) benzene) respectively. The high surface area obtained by this method is equivalent to the maximum value of reported before. Additionally, Yuan [31] confirmed that liquid-assisted grinding could be utilized to construct different MOFs from the same reactant. In the process of grinding terephthalic acid (and zinc or zinc carbonate), adding a small amount of water or methanol or DMF, three different kinds of MOFs can be obtained respectively. Friscic et al. [32] introduced an improved mechanochemical method, which called ion- and liquid-assisted grinding. This method demonstrated that liquid-assisted grinding (LAG) could be utilized to synthesize the MOFs and accelerated such synthesis through nitrate or sulfonate-assisted catalyzed in mechano-synthesis, directing the structure of the product into quadrilateral or hexagonal structure, respectively. Luminescence MOFs also could be synthesized by a mechanochemical method without solvent rapidly and simply [33].

#### 1.2.3. The Sonochemical Method

The sonochemical method was advanced to synthesize MOF-5 primarily [34]. The sonochemical method could shorten the crystallization time of MOFs and make the nucleation homogeneous compared with the traditional solvothermal method [30]. Mg-MOF-74 was synthesized by the sonochemical method for CO_2_ capture and conversion in 2012 [35]. As another example, MOF-177 crystals with high-quality can also be obtained by the sonochemical method. The synthesis time was reduced sharply from 48 h to 30 min with the yield up to 95.6%, while the crystal size only reduced to 5–20 μm. But the adsorption capacity of the crystal is 30 bar higher than that obtained by the solvothermal method [36].

The microwave-assisted solvothermal method is an efficient way to produce nano-sized metal-organic frameworks by microwave irradiation and can obtain new materials in a few minutes [37]. Wu et al. [38] constructed isostructural MOF-74 with both traditional hydrothermal method and microwave-assisted method successfully. It can be found that MOF-74 produced by microwave assisting has a larger specific surface area and micropore volume with more uniform particles. Ren et al. [39] reported that It took only five minutes to synthesize Zr-MOFs using a microwave-assisted modulated method. MOFs still has high crystallinity with clearly visible octahedral shaped crystals.

#### 1.2.4. The Electrochemical Synthesis

The applications of MOFs are generally based on bulk powdered MOFs. To bring their potential into full play in these applications such as sensors, catalysis, separation etc. it’s an ideal choice to use MOF materials as thin films or coatings by electrochemical synthesis [40]. The MOFs were initially developed by the electrochemical synthesis [41], the principle is to give the metal ions of MOF building block by the electrochemical dissolution of anodic to a synthesis solution containing the organic ligand and the conduction salt. The thin films composed by MOF crystals formed by the careful modification of the conditions in electrochemical synthesis.

The common MOF named [Cu_3_(BTC)_2_] composed of Cu(II) ions and the tridentate ligand 1,3,5-benzenetricarboxylic acid (BTC) [42]. Cu(II) ions were imported into the working solution containing BTC and the conduction salt of methyltributylammonium methyl sulfate (MTBS) by applying an anodic voltage to the copper electrode. It was finally found that densely packed films of [Cu_3_(BTC)_2_] crystals are easily prepared by electrochemical synthesis [43]. The well-adhering MOFs films with luminescence can be synthesized by electrochemical synthesis on many electrically conductive solid substrates surface. The poorly luminescent MOFs can be deposited more economically thanks to the partial substitution of metal ions nodes with the improved luminescent performance [44].

### 1.3. The Hybrid Materials Based on MOFs

An easy design and self-assembled method can be conveniently excogitated for the MOFs [45,46,47]. These make MOFs a solid platform for the encapsulation of various guest species to achieve MOFs functionalization [48,49]. Among the functional MOFs, MOFs hybrid materials with luminescence may exhibit various and tunable optical properties [49]. Up to present, the enormous spread of luminescent composition has been embedded into MOFs to merge several novel composite materials, with the united properties better than those of the individual components [50,51,52,53]. The stability matter of luminescent organometallic halide perovskites with high quantum yield limited their availability in different functional applications, Zhang et al. [50] report a two-step synthesis method for perovskite CH_3_NH_3_PbBr_3_ QDs dominated in MOF-5. CH_3_NH_3_PbBr_3_@MOF-5 composites acted out the high strength water resistance and thermal stability improvement, together with better pH adaptability over extensive range compared to naked CH_3_NH_3_PbBr_3_ QDs. Lin et al. [54] synthesized the fluorescent hybrid MOFs by embedding poly-(ethylenimine)-capped carbon quantum dots (CQDs) into the zeolitic imidazolate frameworks (ZIF-8) to develop the chemical sensor. The formed CQDs/ZIF-8 material not only presented high florescent activity and sensing selectivity originated from CQDs but also can enrich the target analytes with high efficiency.

### 1.4. The Harnessing of MOFs and Their Hybrid Materials

MOFs have been used or designed to satisfy the demands of various applications over the last decades. The varied structures and exceptional properties also make MOFs enchanting for possible applications, ranging from gas storage [55] to liquid/ solid-phase separation [56,57], sensing [58], drug delivery [59], heterogeneous catalysis [60] and proton conductivity [61].

The MOFs are a kind of promising adsorbent materials for gas storage due to their structural characteristics [62,63]. Wang et.al [64] investigated the ability of gas storage of BUT-22, a microporous Al(III)-MOF with high stability. BUT-22 exhibited high gas storage capacities for carbon dioxide methane and hydrogen. The MOF-hybrid material was applied to dispersive miniaturized solid-phase extraction (D-µSPE) by ultra-high-performance liquid chromatography. The authors synthesized core-shell shaped SiO_2_@MOF microspheres for the measurement of 14 kinds of polycyclic aromatic hydrocarbons (PAHs) in wastewaters [65]. Jiang [66] reported a magnetic MOF-hybrid material for acarbose detection in rat plasma. This material layer was made from magnetic graphene and Zn-MOFs on both side and was used for acarbose collection in plasma before its qualitative and quantitative by liquid chromatography linked to tandem mass spectrometry (LC-MS/MS). Tian [67] designed the microspheres composed of bimetallic ZnNi-MOF to set up a label-free aptasensor for efficient electrochemical detection of adenosine. The ratio of Zn^2+^ to Ni^2+^ in MOF microspheres was optimized to achieve its best electrochemical activity and binding ability to the aptamer strands. The detection limit of the electrochemical aptasensor was 20.32 fg·mL^−1^ toward adenosine in the linear range of 10^−4^–10^2^ ng·mL^−1^. The synthesis of MOFs was flexible and MOFs had high storage capacities with biocompatibility, which makes MOFs desired materials as drug delivery systems (DDSs) for cancer drugs. CAU-7, a bismuth-based MOF with biocompatibility, was reported for the transportation of sodium dichloroacetate (DCA) and α-cyano-4- hydroxycinnamic acid (α- CHC). CAU-7 could load 33 wt % for DCA and 9 wt % for CHC, slowly releasing DCA for 17 days and CHC for 31 days [68], the result exhibited the therapeutic efficiency of CAU-7 was higher than the free drug approach. It’s a research hotpot that engineering the MOFs to be effective acid-base tandem catalysts. The bimetallic MOFs were synthesized and functionalized by amine to be the acid-base bifunctional catalysts through a fast reflux method and were utilized for tandem reaction. The bimetallic MOFs catalysts exhibit enhanced catalytic activity when compared with the catalyst performance of the monometal based MOFs due to the synergistic effect between two metal ions and the hierarchical pore structure of the bimetallic MOFs [69].

MOFs and their hybrid materials have been also employed as interesting platforms to photovoltaic devices [70] and organic light-emitting devices (OLEDS) [71] thanks to some special properties of the MOF materials with excellent performance and stability. The worldwide need of energy is rapidly increasing day after day, but it should be up against the severe pollution issues of the environment along with decreasing resources in the meantime. The massive research was engineered and executed to solve these important issues. The scholars put great efforts to retrieve such prospect by exploring neoteric and efficient ways to manufacture, transport, keep, and use energy. As the principle of photovoltaic devices transduce photo signals into electric circuits, the most direct tactic to develop the effectiveness of MOFs in photovoltaic applications is applying them as the photoactive materials. The MOFs should be provided with qualified light-harvesting ability range from visible light to near-infrared (NIR) region because the majority photons incident on the surface of the earth originates in this region.

Not only playing an important role in the photoactive materials, MOFs can be conducive to the photovoltaic community by serving as functional additives or interlayers to improve the characteristics and reliability of the derived solar cell devices. The recent major efforts will be introduced into the dye-sensitized solar cells (DSSCs), perovskite solar cells (PSCs), and organic solar cells (OSCs) based on MOFs. Photoanode is a key role in dyes sensitized solar cells (DSSCs). People made much account of gaining the excellent energy conversion efficiency (*η*) from solar energy since the global energy crisis was more and more serious [72,73]. The MOFs are found that lead to a notable enhancement of the specific surface area of photoanodes, which is beneficial for get an efficient dye adsorption capacity. Mesoporous TiO_2_ was derived from the heating treatment of mesoporous polymeric MOFs at 500 °C, the authors used this mesoporous TiO_2,_ to be the photoanodes fabricated with DSSCs, have efficiencies of 8.43% at 100 mW/cm^2^ higher than that of DSSCs with photoanodes of conventional TiO_2_ [74]. He et al. [75] employed the optimized mass fraction of UiO-66 and ZIF-8 with reduced graphene oxide (RGO) to modify photoanode together meanwhile the graphene counter electrode is used to prepare the full-carbon DSSCs. The resulting η was higher than the examples of using RGO/TiO_2_ and pure TiO_2_ photoanodes. Perovskite solar cells (PSCs) were appealing that they had high power conversion efficiency (PCE); rich elemental composition; and cheap, microtherm, and extensible fabricating process. Shen introduced the MOFs (ZIF-8) as an interfacial layer into PSCs for the first time in 2018 [76]. The interfacial layer consisted of ZIF-8 improved the crystallinity and grain sizes of perovskite along with the photovoltaic improvement of the PSCs, leading to a result of a maximum PCE of 16.99%. Co-doped Ti-MOF was prepared by a solvothermal method, using trimesic acid (H_3_BTC) as an organic linker and the node of Co-doped TiO_2_ to the formation of MOFs. The electron transport layer of the Perovskite Solar Cells (PSCs) was made of 1 wt % Co-doped TiO_2_ and the optimum power conversion efficiency was 15.75%, higher than the commercial dyesol TiO_2_ layer of 14.42% [77].

Organic solar cells (OSCs) are a burgeoning technology in recent years and the interfacial layers in OSCs play the key roles in the performances of photovoltaic. The tellurophene-based MOF material was firstly synthesized to be the interfacial layers with high conductivity or charge mobility and enhanced photovoltaic performances [78]. Metal-organic framework nanosheets (MONs) can also be ideal materials for utilization in photovoltaic applications. Sasitharan et al. [79] synthesized the ultra-thin zincporphyrin based MONs with perfect photoelectric characteristics fitted for merging into a polythiophene-fullerene organic solar cell. Apparently, the introduction of MONs into the photoactive layer belong to the photovoltaic device brought about a power conversion efficiency of 5.2%. It was twice as much as the reference devices without MONs, which demonstrated that the MONs as an adjustable class of two-dimensional materials were potential to enhance the function of extensive organic solar cells and other electronic devices.

Organic light emitting diodes (OLEDs) have found favor in market’s eyes on considering the applicability of OLEDs in high resolution display [80,81]. The extensive variety of metal-ligand association as well as the formation of composites, has enabled MOFs to actualize enhanced luminescence performance with serviceability in the field of OLEDs. The luminescent MOFs (LMOFs) and their hybrid materials stood a great chance for the industry of OLEDs with increased potential to solve low efficiency problems due to their enhanced fluorescence lifetimes, large light quantum efficiencies, and high tunability. In recent years, a large number of MOF-involved hybrid materials demonstrated the exceptional performance, MOFs enabled the synergistic effects with the other component of the hybrid materials to the continuation of practical applications [82,83]. LMOFs and their hybrid materials can be regarded as the frontier materials for OLEDs. So far, over 1300 species of LMOFs have been reported, from which much feasibility still hold up to be studied as phosphors for the approaching luminescent devices [84]. The large amount of LMOFs and their hybrid materials have been developed in the recent past as potential candidates for OLEDs application [85,86,87].

### 1.5. The Advantages of Metal-Organic Framework as Fluorescent Sensing Materials

A fluorescence sensor is generally consisted of a sensing unit and a transduction unit to turn the sensed signal into the optical signal when the sensor interacts with the analytes. The important parameters for evaluating chemical sensors include sensitivity, selectivity, material stability and repeatability [88]. Hence, the choice and design of the sensing material are main points concerning sensor application performance. Fluorescence sensors were applied in the measurement of various targets, such as small molecules [89,90], cations [91,92], anions [93,94], and biomolecules [95,96]. The detection of pollutants in the environment and food is crucial because such chemical species pose serious health risks.

The sensing application of MOFs has attracted a large amount of attention in recent years. It has been demonstrated that MOFs show a sizeable potential force for the sensing of temperature [97], pH [98], small molecules [99], solvents and explosives [100]. Porous MOFs sensing materials can effectively adsorb the target and concentrate it in the framework, which improves the sensitivity of detection [101]. MOFs can adsorb molecules selectively depending on the pore size fitting in the analyte size [102]. In addition, the adsorption selectivity can be improved by enhancing the chemical interaction between the adsorbate and the inner surface of MOFs [103]. Analytes detection can be realized based on the MOFs by various way of techniques like mass spectrometry, inductively coupled plasma mass spectrometry (ICP-MS), electrochemical techniques, atomic-absorption spectrometry and atomic-emission spectrometry, etc. [104]. However, the fluorometric sensors stand out from these techniques due to their rapid reaction, easy operation and trace determination of analytes indeed in both solid and liquid phase [105]. For many years, MOFs as fluorometric probes for detection of foodborne [106] and waterborne [107] contaminants have taken attention seriously in recent years.

Moreover, MOFs possess zeolite-like structures and properties, breaking through some limitations of zeolite application in material chemistry:

1. There are many ways to synthesize luminescent MOFs conveniently [108]. Diverse organic ligands and metal nodes of MOFs could recruit optical emission upon excitation with exposure to UV or visible light [109,110]. Additionally, the doping of the luminescent component expanded the application greatly.
2. The organic-inorganic hybrid properties of metal-organic frameworks which containing organic ligands and inorganic metal ions enable coexistence of hydrophilic and hydrophobic channels [111]. The proper pretreatment and sample extraction procedures were indispensable to the traditional detection of food contaminants [112]. The detection sensors based on MOFs and its functional materials could overcome such constraints. The sensors possessed rapid response under practical conditions [113]. Meanwhile, they can realize intelligence, miniaturization, and both qualitative and quantitative analysis [114].
3. MOFs with unlimited active sites can be prone to functionalize with some biological or chemical components [115,116,117,118,119]. The various fabrication strategies, along with the notable luminescence variation for target analytes, enable the luminescent MOFs to be broader applications in monitoring of quality and safety on environment and food.

In this review, we concentrate our attention to the rapid analysis of environmental and food contaminants by fluorometric MOF sensors. A lot of targets viz. heavy metals, persistent organic pollutants, pharmaceuticals, drugs of abuse and pesticides which induce fluorescent response have been discussed briefly in this work.

## 2. The Current States and Progress of MOFs Fluorescence Sensing Application

There are two sensing mechanisms for various targets detection, which called “turn-on” and “turn-off” fluorescent sensors. The addition of target analytes would cause the enhancement of luminescence response in “turn-on” sensors. On the contrary, the central tenet of “turn-off” sensors is to quench the luminescence signal of sensor upon the addition of analytes. Most of the MOFs-sensors were developed via “turn-off’” phenomena [120]. Because the quenching phenomenon can be taken by many species (interfering species) [121]. For the “turn on” method, it can be observed emission enhancement clearly in the dark state compared to the quenching process even in biological systems [122].

### 2.1. Heavy Metal Detection

Pollution of heavy metal poses a severe threat to the environment, food safety and sustainable development of agriculture [44]. Mercury (Hg), Lead (Pb), Copper (Cu), Zinc (Zn) are the toxic heavy metal elements in food and water [123]. The poisonous heavy metals in food are often derived from the enrichment of crops, and contamination during food production, processing, storage and transportation. They can enter the human body through the food chain and have a harmful affection on humankind [124]. It is a potential risk for people because of their long-term accumulation in the human body. Therefore, the accurate detection of trace heavy metal ions is particularly essential, and it’s extremely urgent to establish effective processing samples methods. The highly porous structure of MOFs and their related hybrid materials are reliable to the plasma diffusion of heavy metal ions into their porous structure. The shape and size of the pore plays an important part in determining the selectivity of heavy metal ions adsorption. Therefore, MOFs and their hybrid materials are the ideal adsorbents for extracting heavy metals [125]. For example, Fe_3_O_4_/Cu_3_(BTC)_2,_ growing on the surface of the functionalized Fe_3_O_4_ in-situ, has been widely used in the pretreatment of fish, baby food, vegetables, seafood and agricultural products, to absorb different kinds of heavy metal ions, such as Cd(Ⅱ) [125], Pb (Ⅱ) [126], Cr(Ⅲ) [126], Hg(Ⅱ) [127] et al. It is a smooth fast operation to enrich the heavy metal ions selectively when Cu_3_(BTC)_2_ combined with fire atomic absorption spectroscopy. The composite has high enrichment ability and low detection limit to heavy metal. Additionally, there’s a little time requirements and less solvent consumption for detection.

The development of new fluorescent MOFs has become the focal point to determine heavy metals [128]. Rapid detection using optical measurement based on MOFs have drawn much attention due to its outstanding selectivity and sensitivity. MOFs have been employed or developed as sensing media or probes for kinds of heavy metals which were of the magnetic optical and luminescent properties, such as Hg(Ⅱ) [129], Cu(Ⅱ) [130], and Pb [131], Cr(Ⅵ) [132], etc. It is an exciting idea to synthesize new fluorescent MOFs to detect heavy metals selectively. Luminescent metal-organic frameworks (LMOFs) have been expanded as chemical sensors lately for metal ions detection [133]. Nathan et al. [129] broke through the conventional fluorescence detection strategy. They provided a quantitative fluorescence detection to increase selectivity based on utilizing LMOFs for toxic metal ions, focusing on their heavy metal removal potential in aqueous solution simultaneously.

**Figure 1:**
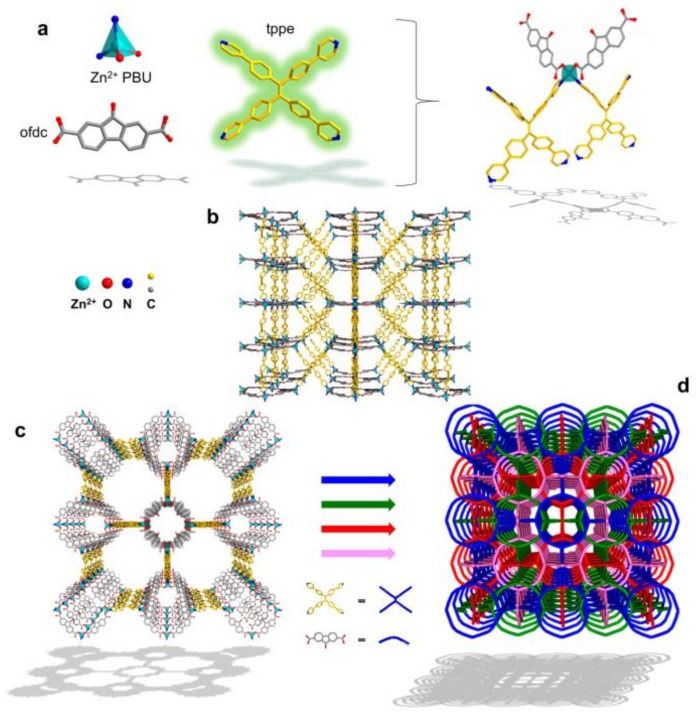
A schematic diagram of LMOF-261 functionalized synthesis and structure. (**a**) The PBU of LMOF-261, displaying a pseudo-tetrahedrally coordinated Zn center bound to two fluorophoric tppe ligands and two ofdc linkers. (**b**) A separate mesh of the LMOF-261 framework observed along the b-axis, containing 1D, edge sharing pentagonal and rhombohedral channels. (**c**) The same mesh displayed downwad the c-axis, showcasing edge sharing octahedral and cylindrical channels. The ofdc linkers point directly into the cylindrical channels extending down the c-axis. (**d**) Simplified LMOF-261 depicting four-fold interpenetration. Each of the three other nets occupies one octahedral pore of the fourth net to create narrow pentagonal channels, sharing edges from multiple nets. Adapted and reprinted with permission from ref [134]. Copyright 2019 American Chemical Society.

As shown in Figure 1, Rudd et al. [134] designed a train of LMOFs with isoreticular shape, combining with an intensely emissive molecular fluorophore and versatile co-linkers into MOF structures, they chose one of these LMOFs named LMOF-263 for Hg^2+^ ions detection. The authors synthesized a robust green luminescent lanthanide metal-organic framework Tb-MOF with 2D network structure. The size of Tb-MOF single crystal can reach to 6 mm, which was rarely reported in kinds of literature. It reveals a first-class recognition ability on detecting Pb^2+^ ion at millimetre level [131]. Liu [135] reported a stable cationic luminescent Eu(III) based metal-organic frameworks (MOFs) and developed a luminescent sensor which displayed instantaneous and selective quenching property of the luminescence toward chromate ions in deionized water and real environmental water samples with high ionic strengths. The scholars reported a kind of stable fluorescent MOF to detect Cr^3+^ in aqueous solutions. These channels of MOFs were filled with cations and exhibited selective adsorption and recyclable detection of Cr^3+^ in aqueous solutions; it all boiled down to the structure and chemical bond of the fluorescent MOFs [136]. Yu et al. [137] reported two imidazole-based Ln-MOFs for Cr_2_O_7_^2–^ or CrO_4_^2–^ detection in water, these MOFs had high fluorescence stability in water more than 30 days. It’s a “turn–off” strategy that the fluorescence of Ln-MOFs was quenched by Cr_2_O_7_^2–^ or CrO_4_^2–^. Du et al. [138] reported a 3D Cd(II) MOFs, constructed abundant carboxylate groups which can bind the metal ions by coordination. Cu^2+^ could quench the fluorescent in water, and the color of MOFs was transformed from yellow to green by visual inspection, which improved the accuracy of the test results.

**Figure 2:**
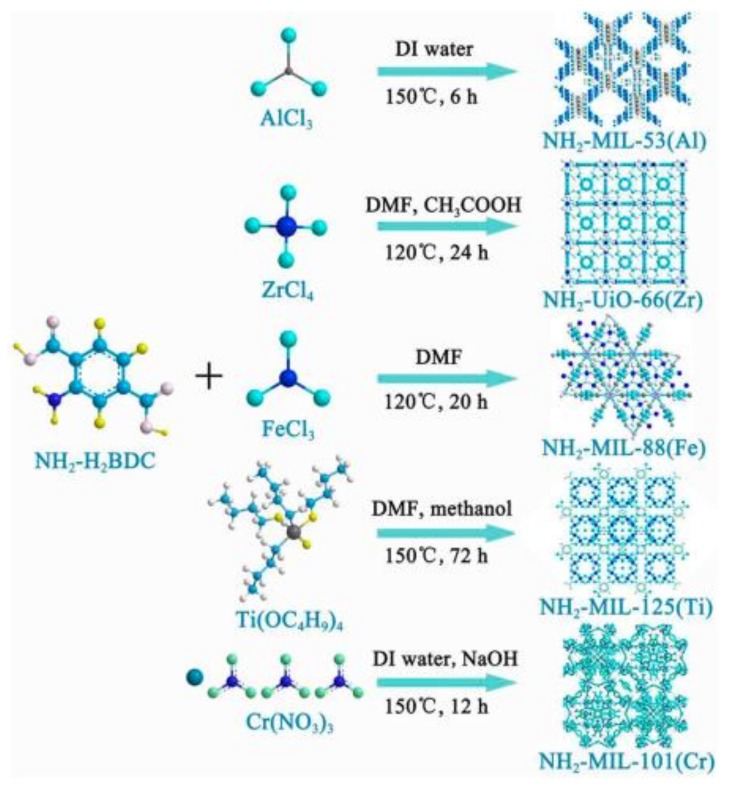
Synthetic routes of the five amino-group luminescent metal-organic frameworks (LMOFs) materials. Adapted and reprinted with permission from ref [139]. Copyright 2016 American Chemical Society.

LMOFs were also modified by chemical groups without complicated synthetic steps. Introduction of the amino group by linkers vested MOFs the fluorescence characteristics. The idea was supported by the production of sorts of amino-group based luminescence MOFs materials with common central metals (Al^3+^, Zr^4+^, Cr^3+^, and Fe^3+^), for Hg^2+^ [139], Cu^2+^ and Pb^2+^ [140] detection. The difference of fluorescence intensity was resulted by changeable degrees of electron transfer efficiency because the various central metals led to these MOF materials different electron acquisition abilities. As shown in Figure 2, the authors synthesized the most fitting fluorescence performance of LMOFs by selecting the suitable high oxidation state center metal. Meanwhile, the strong coordination between amino groups and heavy metals may help amino-based LMOFs improve the adsorption of target ions and achieve detection and removal for heavy metals at the same time [139].

Hao et al. [141] composed a sensory fluorescent MOF for Cd^2+^ detection in aqueous solution. They assembled Eu^3+^ into UiO-66-(COOH)_2_, so that porous structure and servable free carboxyl ligand are with fluorescence able to coordination with Cd^2+^ cations and fluorescent enhancement response.

**Figure 3:**
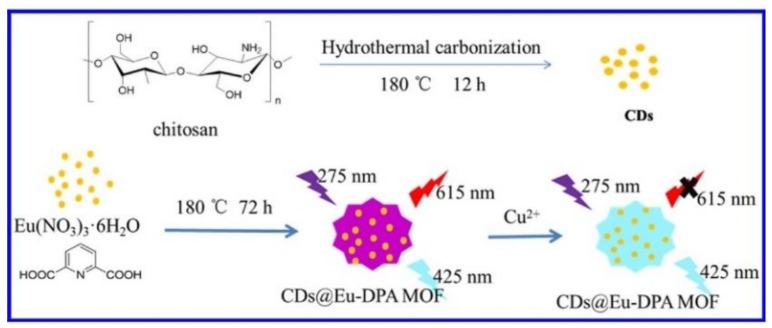
Synthetic route for CDs@Eu-DPA MOFs and the mechanism for Cu^2+^ detection based on the CDs@Eu-DPA MOFs. Adapted and reprinted with permission from ref [148]. Copyright 2017 Elsevier B.V.

The sensors based on LMOFs, lanthanide ions [130,142,143] or aromatic fluorophores [144] doped as their luminescent composition, were growing with every passing day. These sensors are sometimes limited by the low quantum yield (QY) of the inherent luminescence, most of them prefer to emit faint luminescence showing meaningful luminescence response to only a few guests (e.g., acceptors or suitable electron donors) which restricted their further practical use. Desired LMOFs sensory materials expanded towards adhibiting the higher QY guest luminescent materials into MOFs, preserving the original structures and properties of host MOFs generally in the past few years. In 2014, fluorescent carbon quantum dots were encapsulated into zeolitic imidazolate framework materials (ZIF-8) to sense Cu(Ⅱ) ions as the fluorescent probes [54], the hybrid MOF materials enhanced the chemical sensing performance, not only keeping the high fluorescent activity and selectivity originated from CQDs but also accumulating target analytes due to the adsorption property of MOFs. Primarily, metal nanomaterials inset into MOFs can expect improved thermodynamic stability with minimized agglomeration [145,146,147]. In 2017, Hao et al. [148] developed a kind of MOF hybrid material, encapsulating the fluorescent carbon dots (CDs) into Eu-2,6-pyridinedicarboxylic acid (DPA) metal organic frameworks (Eu-DPA MOFs) for the sensitive ratiometric fluorescent detection of Cu^2+^ (Figure 3), Eu-DPA MOFs displayed the uniform nanoscaled ball-flower-like structures so that they were very stable in aqueous solution. The CDs@Eu-DPA MOFs had two kinds of fluorescence emission peaks which derived from CDs and Eu-DPA MOFs. In 2018, Ag NCs were first coated with bovine serum albumin (BSA) and then encapsulated into ZIF-8 to yield the Ag NCs-BSA@ZIF-8 nanocomposites for the fast and sensitive evaluation of Cu^2+^ ions [149].

### 2.2. Pharmaceuticals

The discharged pharmaceuticals and personal care products (PPCPs) or human or veterinary pharmaceuticals (or their metabolites) increased in the environment due to inadequate sewage treatment of wastewater in wastewater treatment plants (WWTPs) [150,151,152]. Owing to the negative effects of these contaminants on the aquatic environment and further affection on food safety, considerable interest has been driven at present on their detection and measurement. MOFs can be the modest adsorbents for the trapping and isolation of human pharmaceuticals and veterinary pharmaceuticals from the aqueous medium because of their water stability and possible abundant binding sites [153,154,155,156,157]. Extensive studies have been published based on the LMOFs for the detection of various species.

#### 2.2.1. Antibiotic Detection

The residues of antibiotics were nothing new in food and environment [158]. Antibiotics are widely used in the treatment and prevention of human or animal diseases because of their strong antibacterial and antivirus effects. The misuse of antibiotics may make them remain in animal food. Antibiotic residues may have long-term detrimental to human health. Additionally, antibiotics will enter the environment with the excretion of people or animals, causing pollution. With regard to the possible adverse effects of human and veterinary antibiotics in the environment and food, the rapid and effective detection of antibiotics is particularly important. At present, the usual methods for the antibiotics detection mainly include high performance liquid chromatography (HPLC) [159], liquid chromatography-mass spectrometry (LC-MS/MS) [160], capillary electrophoresis (CE) [161], enzyme-linked immunosorbent assay (ELISA) [162] and photoelectric/colorimetric sensors [163]. MOFs can be used as a solid-phase microextraction adsorption material to enrich antibiotics in food [164] or water [165]. In 2013, ZIF-8 can be used as solid-phase extractant to establish an on-line solid-phase extraction HPLC method for the determination of oxytetracycline, tetracycline and chlorotetracycline in water and milk samples. The enhancement factors are 35–61 times than other methods. The detection of this method is as low as 1.5–8.0 μg·L^−1^ [166]. In 2016, MOF-5/ILG was constructed for solid- phase microextraction of chloramphenicol and methamphenicol in milk, honey, urine and serum samples [167]. MOF-5/ILG is a kind of metal-organic framework−ionic liquid functionalized graphene nanocomposites (MOF-5/ILG). The MOF-5/ILG composites have good crystal morphology which the covalent bond between the amino group of ILG and the carboxyl group of MOF-5 improves the mechanical stability and structural uniformity of the microcrystalline. The obtained material combines the advantages of MOFs and ILG with high specific surface area (820 m^2^·g^−1^) and good adsorption performance. With the optimal conditions, the method is suitable in virtue of its high precision, high sensitivity and low detection limit (14.8–19.5 ng·L^−1^) with acceptable recoveries of 82.3%–103.2%, and the coating material has good resistance and reusability.

Compared with other strategies, the fluorescent method is more worth applying due to the advantages of convenience and sensitivity. The application of MOFs for antibiotic detection is still in its early stage [168]. Although some advances have been made recently, the experimental schemes are required to applied to practice yet. Tetracyclines (TCs) are one variety of antibacterial substances widely used in the treatment and prevention of animal diseases [169]. The long period of exposure to TCs would cause the increase of the bacterial drug resistance in the human body and harm the health of consumers [170]. In 2018, a new luminescent Zr-MOF was synthesized to set up a dual-functional fluorescent platform [171]. This platform can both detect and remove tetracycline in water with high sensitivity. TCs could cause fluorescent quenching of luminescent Zr-MOF and could be removal efficiently due to metal-ligand bonding between Zr_6_ nodes and tetracycline. The adsorption amount (*Qm*) of TC in Zr-LMOFs is up to 423 mg·g^−1^, and the LOD is lower as 30 nM. Gao [172] developed a platform for the fluorescence detection and degradation of tetracycline simultaneously. This dual-function platform was relying on a p-type semiconductor@MOFs (CuBi_2_O_4_@ZIF-8), combining the p-type semiconducting own catalytic property of CuBi_2_O_4_ with the distinctive porous nanostructure and stability of ZIF-8. Tetracycline could enhance the CuBi_2_O_4_@ZIF-8 remarkably and the detection level was 26 nM.

In 2019, Liu et al. [173] reported the in-sbdc MOF with strong and stable emission in water for tetracycline type antibiotics analysis in environment and food. Chlortetracycline (CTC) and oxytetracycline (OTC) were chosen on behalf of tetracycline type antibiotics due to their extensive application. The quantum yield of this MOFs was 13%. A series of tetracyclines could “turn off” the fluorescent emission through an inner filter effect with the detection limit of 0.28–0.30 μM. The sensor could practice the application of the detection of contaminants in both water and food.

Accordingly, Li et al. reported a new lanthanide MOFs (Ln-MOFs) based sensor constructed to a “turn off” detection method for metronidazole (MDZ) [174]. The logical design of functional LMOFs requires a reasonable selection of the ligands with distinctive structures and predominant features [175]. The semi-rigid polycarboxylate ligands play a dominant part in the red luminescence of MOFs with the high quantum yields of 75.57%. Designing the technology of the multi-selective luminescence sensors to distinguish a series of compounds is very important and challenging [176,177]. White-light-emitting decoding sensing based on Ln-MOFs is an up-and-coming candidate for the multi-selective application of luminescence sensing. Yu et al. [178] synthesized three isomorphic Ln-MOFs by solvothermal reactions with red, blue, and green emission, respectively. The white-light-emitting complex materials acted as a handy utility luminescent platform combined with a novel method of 3D decoding map for differentiating eight popularly-used antibiotics. Metronidazole (MDZ), dimetridazole (DTZ), and ornidazole (ODZ) were distinguished from other five antibiotics through luminescence quenching processes.

Nitrofuran antibiotics (NFAs) are efficient for the treatment of bacterial and protozoan infections in human, animal husbandry and fisheries and aquaculture [179,180,181,182]. The authors [182] designed a chemical sensor based on a thin-film composed of the Ln-MOFs coated on a stainless steel wire mesh (SSWM) combined with Co_3_O_4_ nano-anchor fixation method for the NFAs detection. The NFAs could quench the LMOF thin film, facilitate a three-dimensional porous, flexible, and processable “turn-off” sensor. They also developed a Ln-MOFs based sensor for NFAs detection in the same year (2017) [183]. They reported an anti-interference capability enhanced luminescent sensor of Ln-MOF filled mixed matrix membranes (MMMs) based on inner filter effect. Zhu et al. [184,185] have reported two kinds of LMOFs for NFAs in 2019 and 2020. The synthesized LMOFs can be easily prepared, and the methods used to NFAs detection were outstanding stability (at least ten cycles).

#### 2.2.2. Hormones

Estrogens were female reproductive hormones resulted from the excretion of human and animal, plants, or fungi. These sources of estrogen existed in the environment. These compounds are responsible for the development of female secondary sex characters and the health benefits of women, including the prevention of heart disease and osteoporosis [186]. They are also used for hormone therapy and oral contraceptives [187,188,189]. Estrogens can offer the risks because they act as endocrine disruptors (EDCs) resulting in fish feminization [190] and influencing plant or human health. Estrogens are excreted through urine, primarily [191]. The investigation of estrogens and their analogues in human urine has become indispensable for the health of women due to the above reasons.

In the initial reports, some analytical methods based on chromatography have been proposed to measure estrogens in urine samples and other biological samples [192,193,194,195,196,197]. However, due to the trace levels of estrogens in real samples, and the massive complexity of these matrixes [198], which can block compatibility with the analysis instrument, an excellent sample preparation material is useful before HPLC analysis of estrogens. Ragab et al. [199] reported functional ZIF-8 modified by a polytetrafluoroethylene (PTFE) double layer microfiltration membrane to test the removal capacity of micropollutants from water. Gao et al. [200] reported a vortex-assisted membrane extraction (VA-ME) material consisted of some metal-organic framework mixed-matrix membranes (MOF-MMMs), applying for the measurement of four estrogens in human urine. The material and detection method were handy practical and suitable for an enormous spread of MOF materials and retained the embedded MOFs original properties. Huang [201] reported a one-pot method to synthesize the magnetic MIL-101 composites As magnetic solid-phase extraction materials for preconcentration of estrone (E1), 17β-estradiol (E2), estriol (E3) and × (BPA) within the range of 0.2–100 μg L^−1^ and low detection limits of 0.06–0.22 μg L^−1^.

The MOFs named Fe-MIL-88B–NH_2_ could absorb the DNA aptamer, a fluorescent BPA “turn-on” biosensor was designed based on this phenomenon [202]. In this system, the fluorescent dye labelling aptamer used as the sensing recognition element, adsorbed by Fe-MIL-88B–NH_2_ in aqueous solution, and the fluorescence of the dye was quenched by MOFs. BPA would combine with the fluorescent DNA in the sample solution, leading to the fluorescent DNA probe release from Fe-MIL-88B–NH_2_ and turning on the fluorescence. The detection limit was 4.1 × 10^−14^ mol L^−1^, and the linear working range was 5.0 × 10^−14^–2.0 × 10^−9^ mol L^−1^ with good selectivity.

### 2.3. Pesticides

To increase crop yield and reduce the infection of pests and diseases, crops can be sprayed with insecticides, herbicides and foliar chemicals in excess. This leads to pesticide residues accumulating through the food chain enrichment. Therefore, more and more attention has been paid to the monitoring and detection of pesticide residues. Organophosphorus, organochlorine, carbamate, pyrethroid and organometallic pesticides are the conventional residual pesticides in food. The routine detection of agricultural residues needs to go through the steps of sample preparation, purification and enrichment, separation and detection, and comprehensive analysis, especially the sample processing is complicated. Highly efficient and rapid sample pretreatment and detection technology have become an urgent problem in agricultural residue analysis.

UiO-66-NH_2_ was used as an efficient adsorbent to disperse CPAHs in vegetable samples by micro solid-phase extraction. It is pointed out that the adsorption of UiO-66-NH_2_ on CPAHs is mainly based on the ionic bond between the amino group of MOF and carboxyl group of CPAHs [203].

In recent years, LMOFs was used to determine pesticides in food. The principle of luminescent sensing for pesticide is the interaction between the electron absorbing NO_2_ group of organophosphate pesticides (Ops) and electron-rich centers of MOFs. This mechanism serves as a basic prop of theory for the “turn-off” luminescent intensity of both MOF quenching sensors. Pawan Kumar explores the utility of luminescent MOF-5 for the direct chemosensing of food nitro Ops [204]. The synthesized MOF-5 has a good fluorescence property, which provides a direct chemical sensor for four kinds of Ops. The linear of the above Ops detection is in the concentration range of 5–600 ppb separately. The proposed method is also suitable for the detection of malathion, dichlorvos and monocrotophos. This research group also reported another luminescent nanocrystal metal–organic framework (NMOF1) to detect Ops in the same year. This material has brought down the detection limit to Ops (1ppb) [112].

**Figure 4:**
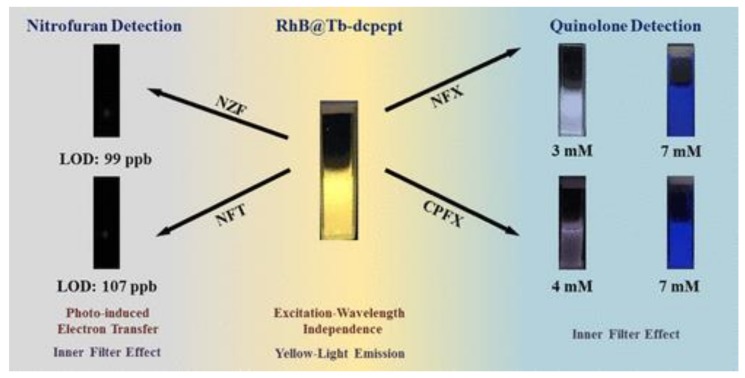
The diagram of the sensor for Cu^2+^ detection based on the RhB@Tb-dcpcpt. Adapted and reprinted with permission from ref [206]. Copyright © 2019 American Chemical Society.

In 2017, Zhu et al. [205] reported a “turn-off” chemical sensor with highly efficient and quick responsive, using a Zn(II)-based 3D fluorescent MOF denoted FCS-1, was used to detect a series of sulfonamide antibiotics in the simulated waste water. FCS-1 with highly ordered structure revealed stable in the wide pH range of wastewater (pH = 3.0–9.0). FCS-1 was quenched effectively through photoinduced electron transfer reaction by sulfonamide antibiotics with low detection limits. RhB@Tb-dcpcpt [206] is a kind of host−guest MOF composite, capturing Rhodamine B into the channels of Tb-dcpcpt via an ion-exchange process. The composite exhibits stable dual luminescence of RhB and Tb^3+^ ions, nitrofuran antibiotics were detected by luminescent quenching process and quinolone antibiotics were detected by luminescent color-changing process. The detection limit is as low as 99 ppb for nitrofuran antibiotics, and the visual LOD is low to 4mM for quinolone antibiotics (Figure 4).

### 2.4. Persistent Organic Pollutants

Persistent organic pollutants (POPs) are a class of synthetic or natural compounds, which can have long distance migration through environmental media and long-term existence in the environment [207]. POPs are concerned as highly toxic, which should not be biologically treated directly [208]. Even if a trace exists in the environment or food, it will pose huge potential hazards to human health because of its bioaccumulation. Owing to its harmfulness and trace residual amounts, it is essential to carry out the sensitive and convenient techniques for the analysis and detection of POPs. There are many types of chemical compounds belonging to POPs, such as persistent toxic substances (PTS), polycyclic aromatic hydrocarbons (PAHs), phenols, and so on [209]. In recent years, MOFs and its functional materials have been used in the pretreatment of food [210] and water samples [211] containing POPs

To most POPs, there are lots of structures in common, so that they can be removed by the same material through an efficacious fashion. POPs may have one aromatic ring at least in the fabric so that these aromatic POPs provide an electron-rich environment for adsorption or reaction due to π-π conjugation [212]. Therefore, MOFs with a large amount of the coordinatively unsaturated sites (CUS), can with advantage adsorb the POPs through the coordination effect of lone pair of electrons.

Porous MOFs are more suitable for the removal and detection of various analytes in aqueous media [213]. The specific surface area of MOFs is not the only decisive element to the adsorption efficiency but also combined with each side such as adsorption affinity or sensitivity between POPs and MOFs. Stability in the matrix is another excellent performance for the rational and useful application. For example, the composites of MIL-68(Al) [214] and MIL-53(Cr) [215] were doing very well in the adsorption of phenol and bisphenol-A (BPA). The dominant factors were hydrogen bonding and π-π conjugation between MOFs and analytes.

LVMOF-1 [216] is a kind of 3D MOFs with the ability to sense electron-rich benzene derivatives. LVMOF-1 has outstanding chemical stability to enable color and luminescence responsive to phenol synchronously. The bimodal sensors based on electron-deficient channels of MOFs are classy selective against a large number of other organic interfering substance. The detection limits ranged from 1 to 9 μg L^−1^.

Ning et al. [217] considered the backbone structure of MOFs would affect the sensing response of the chemical sensor. They designed three different pyrene types MOFs to investigate the potential of fluorescent MOFs for PAHs detection. This work demonstrated the topological structure of MOF could affect the variation of excited-state energy of the material represented sensing performance, and the detection limit of the fluorescent sensor was low to ng·L^−1^ level.

### 2.5. Other Hazardous Substances

#### 2.5.1. Mycotoxins

Food spoilage fungi are universal contaminants of food raw materials [218]. Their activity and colonies contents are decided in light of the general environmental conditions and the nutrients of the food substrate. Under the conducive growth ecological conditions, these fungi can emerge as extensive secondary metabolites. Some of these metabolites have toxicity and a major influence if they enter the environment and animal food chains. Mycotoxins are stable toxic secondary metabolites produced by fungi [219]. They can enter human and animal bodies through feed or food and play their biological effects, starting a toxic chain for human beings [220]. Mycotoxins are of particular vital as they are very heat stable and difficult to destroy [221]. It is an urgent need to monitor the mycotoxins in human foods and animal feeds to guarantee the safety of food. Additionally, the fluorescent detection method for mycotoxin is convenient and cost-effective with a significant influence on food safety in the world.

**Figure 5:**
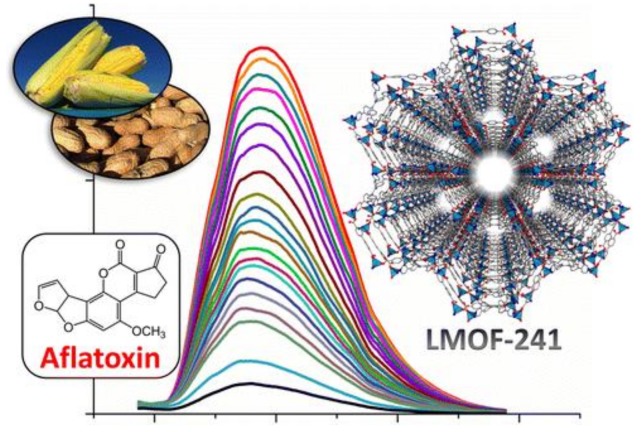
The diagram of Aflatoxin B1 detection using LMOF-241 (The inner is the structure of LMOF-241, the color curves in the caption were emission spectra of LMOF-241 with the incremental addition of AFB1). Adapted and reprinted with permission from ref [225]. Copyright © 2015 American Chemical Society.

Aflatoxins are derivatives of dihydrofuran coumarin with similar chemical structures and common carcinogen [222], which can be found in food matrix supporting fungal growth [223]. Aflatoxin B1 is the most toxic and the most popular mycotoxin encountered [224]. Hu [225] designed a new three-fold interpenetrated luminescent MOF (LMOF-241) with blue light emission as shown in Figure 5. The channels of diameter along c-axis were ~16.6Å which allowed Aflatoxin B_1_ to enter the pores. The maximum emission intensity of LMOF-241 could be quenched extremely by AFB_1_ at 46 ppb, lower than the AFB_1_ tolerant level of 300 μg L^−1^.

Li [226] reported another LMOF named ZrCAU-24 to detect AFB_1_ without specificity modifications. The LMOF is high stability in water and acute fluorescence quenching containing AFB_1_. The detection limit reaches as low as 19.97 μg L^−1^, and the responses of other common biotoxins were all significantly below that of AFB_1_. Ochratoxin A (OTA) is a frequent natural contaminant of a lot of food. Fe_3_O_4_/g-C_3_N_4_/HKUST-1 was firstly synthesized and fabricated as a “turn on” fluorescent biosensor for ochratoxin A (OTA) [227]. Fe_3_O_4_/g-C_3_N_4_/HKUST-1 can wholly quench the fluorescence of the post-dye aptamer because of its electron strong adsorption capacity. There has been selective binding between aptamer and OTA with unusual affinity, causing the releasing of the dye-labelled aptamer from the Fe_3_O_4_/gC_3_N_4_/HKUST-1 and therefore results in the recovery of fluorescence. The detection range of the fluorescent sensor was within 5.0–160.0 ng·mL^−1^, and the LOD is 2.57 μg L^−1^ (S/N = 3).

#### 2.5.2. Amines

It is urgent to sense unhealthy small molecules, as even existence at trace concentration may harm the human health [228,229]. Furthermore, there is a pressing need for detection of amines in the assessment of food safety and diseases prevention and control [230]. Mallick et al. [231] reported a “turn-off” sensor based on the luminescent Zr-BTDB-fcu-MOF with ultrasensitive detection for amines as low as 66 nM. This ultrasensitive detection is according to hydrogen bonding between the Zr-MOF and the amines, the amines could turn-on the luminescence and enhance the emission intensity.

Aromatic amines are infamous environmental pollutants which are often released into the environment from contemporary industries along with wastes and from the resolution of azo-dyes in some kinds of daily used things [232]. The exposure to aromatic amines is suspected of causing carcinogenicity through ingestion, inhalation and skin contact under shallow doses. Therefore, the assessment of their content in environmental waters is increasingly vital for the security of health and the environment. There has been an extensive interest shown in the recognition and sensing of aromatic amines because of their essential roles in biological and environmental systems. The sensitive fluorescent analytical method based on two multifunctional MOFs was reported for the rapid and efficient detection of aromatic amines (the “turn-on” and “turn-off” sensing materials) [233]. The fluorescent experiments show that the two MOFs can be quenched selectively by p-nitroaniline with high sensitivity. Diphenylamine showed the most distinct enhancement effect than those of other given aromatic amines under the same experimental conditions. The corresponding authors were reported another two multifunctional Mn(II) MOFs for the sensing of aromatic amines. This describes that the MOFs had great potential in detection applications. Aniline and its derivative products (e.g., nitroanilines) are very toxic to human beings [229,234]. In 2015, Guo [235] reported a luminescent terbium metal–organic framework with the ketone groups functionalization for the sensing of aniline selectively and sensitively. The LMOFs has the advantages of good absorbability and heat stability, the ketone groups on the 1D channel surfaces could give them specific adsorption ability for binding the guest molecules. In 2016, two isostructural lanthanide MOFs were synthesized for anilines and Fe^3+^ detection, and the fluorescent ligand H_2_L on organic linkers enhanced the fluorescent emissions of the lanthanide MOFs. Among the analytes, diphenylamine significantly enhanced the fluorescence, and p-nitroaniline and Fe^3+^ could quench this. The development of this fluorescent MOFs-sensor identified as appropriate for the development of multifunctional sensors (both “turn-on” and “turn-off”).

## 3. Future Perspectives and Concluding Remarks

In this review, we have briefly outlined the MOFs synthesis and advantages mainly on the fluorescent sensing application for the detection of various analytes in the environment and food. The detection results summarized in this work were listed in Table 1.

We introduced the types of analytes from heavy metal ions to toxic organic pollutants which results in a fluorescence emission response of LMOFs and MOFs involved hybrids. The fluorometric sensors based on MOFs are still very much belonging to the “turn-off” mechanism, a few sensors are based on the “turn-on” mechanism. Given the broad spectrum of toxic and harmful substances that have the critical impacts on food and environment safety, it is the top priority to design more sensors based on luminescence MOFs. The research on MOF materials has made remarkable progress in recent years, especially in the detection of harmful substances in food. To comprehensively evaluate the current research situation, the research on MOF materials needs to be emphasized from the following aspects:

1. Raising the water stability and reusability of LMOFs. This can be achieved by introducing high valence metallic metals and multi-dental organic ligands into LMOFs.
2. Enhance the fluorescence stability of LMOFs to improve the performance of targets detection, particularly.
3. For improving adsorption efficiency for the range of quantitative phase analysis of targets: (i) the precise control for the channel of LMOFs, where the pore diameter is fitting the molecular dimension of targets; (ii) the improvement of pore affinity, which can be tuned by adjusting the organic ligands to increase the number of electrophilic groups of to bind the targets; (iii) increase hydrogen bond binding sites, which can be enhanced by introducing oxygen-containing functional groups on MOFs to bind targets.

In a short, the MOF-based fluorometric sensors can open up a new direction toward the chemo-sensing of food and environment relevant analytes, and also applications of bio-detection of microorganisms and cells. Therefore, continuous efforts will continue in this regard.

## Figures and Tables

**Table 1:** The application based on LMOFs and their hybrid materials in sensors for the safety of food and environment.

Material	Target	Strategy	Response Range	Detection Limit/Stern- Volmer Constant (Ksv) M^-1^	Ref
MIL-101-NH_2_	Cu^2+^, Pb^2+^	turn-off	10–1000 μM	~1.8 μM	[140]
CQDs/ZIF-8	Cu^2+^	turn-off	2–1000 nM	80 pM	[54]
[Cd_3_(L)_2_(H2O)_5_] · (H_2_O)_4_	Cu^2+^	turn-off	0–100 μM	3.65 × 10^4^ M^−1^	[138]
Eu_2_(FMA)_2_(OX) (H_2_O)_4_·4H_2_O	Cu^2+^	turn-off	-	528.7 M^−1^	[130]
CDs@Eu-DPAMOFs	Cu^2+^	turn-off	50 nM–10 μM	26.3 nM)	[148]
AgNCs-BSA@ZIF-8	Cu^2+^	turn-off	2.0 × 10^-4^–80.0 μM	0.05 nM	[149]
NH_2_-MIL-53(Al)	Hg(Ⅱ)	turn-off	1–17.3 μM	0.15 μM	[139]
LMOF-263	Hg^2+^	turn-off	-	~3.3 ppb	[129]
Tb-MOF	Pb^2+^	turn-off	8 × 10^−^^4^–3.4 × 10^−^^7^ M	10^-7^ M	[131]
[Eu_7_(mtb)_5_ (H_2_O)_16_]·NO_3_·8DMA·18H_2_O	Cr(VI)	turn-off	1 ppb–300 ppm	0.56–1.75	[132]
[Me_2_NH_2_]_4_[Zn_6_ (qptc)_3_(trz)_4_]· 6H_2_O	Cr(III)	turn-off	0–40 μM	4.39 × 10^4^ M^−1^	[136]
Ln-MOFs	Cr_2_O_7_^2−^, CrO_4_^2−^	turn-off	-	1.915 × 10^4^ M^−1^, 1.141 × 10^4^ M^−1^	[137]
UiO-66(Zr)–(COOH)_2_	Cd^2+^	turn-on	0–500 μM	0.06 μM	[141]
PCN-128Y	TCs	turn-off	0–0.9 μM	30 nM	[171]
CuBi_2_O_4_@ZIF-8	TC	turn-on	0–45 μM	26 nM	[172]
In-sbdc	CTC, OTC	turn-off	-	0.28–0.30 μM	[173]
[Eu_2_(2,3΄-oba)_3_(phen)_2_]_n_	MDZ	turn-off	0.06–0.17 mM	2.75 μM	[174]
Eu/Gd/Tb-dcpcpt	TC NZF NFT SDZ CBZ MDZ DTZ ODZ	turn-on; turn-off	0–100 μM	0.887 ppm 2.770 ppm 0.189 ppm 1.890 ppm 0.373 ppm 0.217 ppm 0.219 ppm 0.142 ppm	[178]
Eu-BCA thin-film	NFAs	turn-off	0–40 μM	~0.16 μM	[182]
Tb-AIP MMMs	NFAs	turn-off	0–60 μM	~ 0.30 μM	[183]
{[Tb-(TATMA) (H_2_O) · 2H_2_O}n	NFAs	turn-off	0–5 μM	up to 3.35 × 10^4^ M^−1^	[184]
{[Cd_3_(TDCPB)·2DMAc]·DMAc·4H_2_O}_n_	NFAs	turn-off	0–100 μM	up to 7.46 × 10^4^M^−1^	[185]
Fe-MIL-88B–NH_2_	BPA	turn-on	5.0 × 10^−14^ –2.0 × 10^−9^ mol L^−1^	4.1 × 10^−14^ mol L^−1^	[202]
MOF-5	nitro OPs	turn-off	5–600 ppb	5 ppb	[204]
FCS-1	sulfonamide antibiotics	turn-off	-	1.87 × 10^3^- 3.7 × 10^4^ M^−1^	[205]
RhB@Tb-dcpcpt	NZF; NFT NFX; CPFX	turn-off	0–0.1 mM	99 ppb; 107 ppb; 69 ppb; 53 ppb	[206]
LVMOF-1	Phenol; aniline; benzenediols; aminophenols	turn-off	0–4 μM	1–9 μg L^−1^	[216]
NU – 1000	acenaphthylene; pyrene; fluoranthene	turn-on	0–16 μg L^–1^; 0–8 μg L^–1^; 0–10 μg L^–1^	183 ng L^–1^; 40 ng L^–1^; 35 ng L^–1^	[217]
LMOF-241	aflatoxin B_1_	turn-off	0–5.0 × 10^−6^ M	46 ppb	[225]
Zr-CAU-24	aflatoxin B_1_	turn-off	0.075–25 mM	19.97 ppb	[226]
Fe_3_O_4_/g-C_3_N_4_/HKUST-1	ochratoxin A	turn-on	5.0–160.0 ng mL^−1^	2.57 ng/mL	[227]
Zr-BTDB-fcu-MOF	methylamine; aniline	turn-on; turn-off	(0.5–5) × 10^−6^ M (3–18) × 10^−6^ M	66.2 nM 160 nM	[231]
